# 15-Hydroxy­ethyl-19-isopropyl-5,9-dimethyl-14,16-dioxo-15-aza­penta­cyclo­[10.5.2.0^1,10^.0^4,9^.0^13,17^]nona­dec-18-ene-5-carboxylic acid

**DOI:** 10.1107/S1600536809032954

**Published:** 2009-09-12

**Authors:** Xu Xu, Zhan-qian Song, Shi-bin Shang, Hong-xiao Wang, Xiao-ping Rao

**Affiliations:** a Institute of Chemical Industry of Forest Products, Chinese Academy of Forestry, Nanjing 210042, People’s Republic of China

## Abstract

The title compound, C_26_H_37_NO_5_, which was synthesized from monoethano­lamine and maleopimaric acid, consists of two fused and unbridged cyclo­hexane rings. They form a *trans* ring junction with a chair conformation. The two methyl groups are in axial positions. In the crystal, inter­molecular O—H⋯O hydrogen bonds link adjacent mol­ecules into a layer structure. Two C—H⋯O interactions are also present.

## Related literature

For the synthesis of maleopimaric acid derivatives, see: Walter & Ray (1967 ▶). For the use of the title compound in varnishes and surface coatings, see: Penczek (1970 ▶); Xiao (2003 ▶).
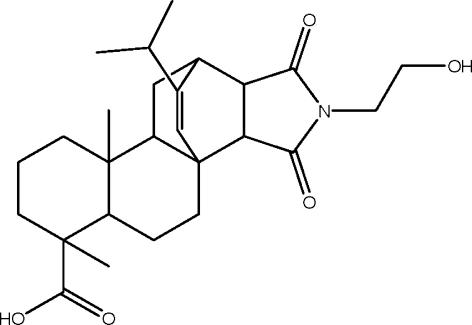


## Experimental

### 

#### Crystal data

C_26_H_37_NO_5_
*M* *_r_* = 443.57
Monoclinic, 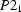

*a* = 12.274 (3) Å
*b* = 6.9550 (14) Å
*c* = 14.445 (3) Å
β = 102.22 (3)°
*V* = 1205.2 (4) Å^3^
*Z* = 2
Mo *K*α radiation
μ = 0.08 mm^−1^
*T* = 293 K
0.30 × 0.20 × 0.10 mm

#### Data collection

Enraf–Nonius CAD-4 diffractometer
Absorption correction: ψ scan (North *et al.*, 1968 ▶) *T*_min_ = 0.975, *T*_max_ = 0.992
2487 measured reflections
2373 independent reflections
1744 reflections with *I* > 2σ(*I*)
*R*_int_ = 0.024
3 standard reflections every 200 reflections intensity decay: 1%

#### Refinement

*R*[*F* ^2^ > 2σ(*F* ^2^)] = 0.074
*wR*(*F* ^2^) = 0.189
*S* = 1.00
2373 reflections
271 parameters
22 restraints
H-atom parameters constrained
Δρ_max_ = 0.44 e Å^−3^
Δρ_min_ = −0.33 e Å^−3^

### 

Data collection: *CAD-4 EXPRESS* (Enraf–Nonius, 1994 ▶); cell refinement: *CAD-4 EXPRESS*; data reduction: *XCAD4* (Harms & Wocadlo, 1995 ▶); program(s) used to solve structure: *SHELXS97* (Sheldrick, 2008 ▶); program(s) used to refine structure: *SHELXL97* (Sheldrick, 2008 ▶); molecular graphics: *ORTEP-3* (Farrugia, 1997 ▶); software used to prepare material for publication: *SHELXTL* (Sheldrick, 2008 ▶).

## Supplementary Material

Crystal structure: contains datablocks I, global. DOI: 10.1107/S1600536809032954/ng2627sup1.cif

Structure factors: contains datablocks I. DOI: 10.1107/S1600536809032954/ng2627Isup2.hkl

Additional supplementary materials:  crystallographic information; 3D view; checkCIF report

## Figures and Tables

**Table 1:** Hydrogen-bond geometry (Å, °)

*D*—H⋯*A*	*D*—H	H⋯*A*	*D*⋯*A*	*D*—H⋯*A*
O2—H2c⋯O5^i^	0.85	2.16	3.010 (9)	178
O5—H5a⋯O4^ii^	0.85	2.34	3.076 (10)	145
C10—H10*A*⋯O3^iii^	0.98	2.55	3.470 (6)	157
C14—H14*A*⋯O3^iii^	0.98	2.38	3.316 (7)	159

Symmetry codes: (i) 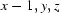; (ii) 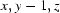; (iii) 
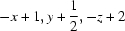
.

**Fig. 1.**
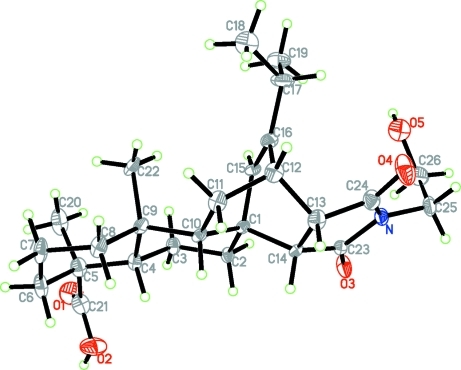
A view of the molecular structure of the title compound, showing displacement ellipsoids at the 15% probability level.

**Fig. 2.**
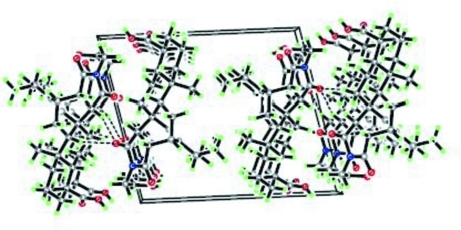
A view of the packing of the title compound.

## supplementary crystallographic information

### Comment

Maleopimaric acid is a readily obtainable compound, which is made by the reaction of maleic anhydride and rosin. A number of new derivatives of maleopimaric acid have been prepared (Walter, 1967). The title compound is one of modified products of maleopimaric acid, which could be used in varnishes and surface coatings (Xiao, 2003). Although, it has been prepared by Penczek P. (Penczek, 1970), the crystal structure of it has not yet been reported. In this work, we describe the crystal structure of the title compound. The molecular structure is shown in Fig. 1 and the crystal packing in Fig.2.

### Experimental

Maleopimaric acid (80.0 g) and monoethanolamine (35.8 g) were slowly heated to 130 degrees centigrade and the reaction was carried out for 2 h. Subsequently, the resulting acrylic modified rosin was cooled to room temperature. Then acetone (150 ml) was added dropwise successively with constant stirring. After dropping the mixture was stirred for another 15 minutes and then filtered. The title compound was precipitated from the solution. Crystals of the title compound suitable for X-ray diffraction were obtained by slow evaporation of an ethanol solution. The crystal data were collected on an Enraf–Nonius CAD-4 difractometer. Data collection and cell refinement were performed using Enraf–Nonius *CAD-4 Software*.

### Refinement

All H atoms bonded to the C atoms were placed geometrically at the distances of 0.93–0.98 Å and included in the refinement in riding motion approximation with *U*_iso_(H) = 1.2 or 1.5*U*_eq_ of the carrier atom.

### Crystal data

**Table:**

C_26_H_37_NO_5_	*F*(000) = 480
*M**_r_* = 443.57	*D*_x_ = 1.222 Mg m^−^^3^
Monoclinic, *P*2_1_	Mo *K*α radiation, λ = 0.71073 Å
Hall symbol: P 2yb	Cell parameters from 25 reflections
*a* = 12.274 (3) Å	θ = 9–12°
*b* = 6.9550 (14) Å	µ = 0.08 mm^−^^1^
*c* = 14.445 (3) Å	*T* = 293 K
β = 102.22 (3)°	Block, colorless
*V* = 1205.2 (4) Å^3^	0.30 × 0.20 × 0.10 mm
*Z* = 2	

### Data collection

**Table:**

Enraf–Nonius CAD-4 diffractometer	1744 reflections with *I* > 2σ(*I*)
Radiation source: fine-focus sealed tube	*R*_int_ = 0.024
graphite	θ_max_ = 25.2°, θ_min_ = 1.4°
ω/2θ scans	*h* = 0→14
Absorption correction: ψ scan (North *et al.*, 1968)	*k* = 0→8
*T*_min_ = 0.975, *T*_max_ = 0.992	*l* = −17→16
2487 measured reflections	3 standard reflections every 200 reflections
2373 independent reflections	intensity decay: 1%

### Refinement

**Table:**

Refinement on *F*^2^	Primary atom site location: structure-invariant direct methods
Least-squares matrix: full	Secondary atom site location: difference Fourier map
*R*[*F*^2^ > 2σ(*F*^2^)] = 0.074	Hydrogen site location: inferred from neighbouring sites
*wR*(*F*^2^) = 0.189	H-atom parameters constrained
*S* = 1.00	*w* = 1/[σ^2^(*F*_o_^2^) + (0.09*P*)^2^ + *P*] where *P* = (*F*_o_^2^ + 2*F*_c_^2^)/3
2373 reflections	(Δ/σ)_max_ < 0.001
271 parameters	Δρ_max_ = 0.44 e Å^−^^3^
22 restraints	Δρ_min_ = −0.33 e Å^−^^3^

### Special details

**Table:**

Geometry. All e.s.d.'s (except the e.s.d. in the dihedral angle between two l.s. planes) are estimated using the full covariance matrix. The cell e.s.d.'s are taken into account individually in the estimation of e.s.d.'s in distances, angles and torsion angles; correlations between e.s.d.'s in cell parameters are only used when they are defined by crystal symmetry. An approximate (isotropic) treatment of cell e.s.d.'s is used for estimating e.s.d.'s involving l.s. planes.
Refinement. Refinement of *F*^2^ against ALL reflections. The weighted *R*-factor *wR* and goodness of fit *S* are based on *F*^2^, conventional *R*-factors *R* are based on *F*, with *F* set to zero for negative *F*^2^. The threshold expression of *F*^2^ > σ(*F*^2^) is used only for calculating *R*-factors(gt) *etc*. and is not relevant to the choice of reflections for refinement. *R*-factors based on *F*^2^ are statistically about twice as large as those based on *F*, and *R*- factors based on ALL data will be even larger.

### Fractional atomic coordinates and isotropic or equivalent isotropic displacement parameters (Å^2^)

**Table:**

	*x*	*y*	*z*	*U*_iso_*/*U*_eq_	
N	0.7569 (3)	0.2774 (8)	0.9683 (3)	0.0527 (12)	
C1	0.4892 (4)	0.2559 (8)	0.8217 (3)	0.0363 (11)	
O1	0.0325 (6)	−0.1069 (12)	0.7369 (5)	0.123 (2)	
C2	0.4169 (4)	0.1064 (8)	0.8588 (3)	0.0423 (12)	
H2A	0.3918	0.1602	0.9126	0.051*	
H2B	0.4622	−0.0055	0.8807	0.051*	
O2	0.0550 (5)	0.1493 (10)	0.8226 (4)	0.108	
H2C	0.0039	0.0929	0.8439	0.162*	
O3	0.6287 (3)	0.0996 (9)	1.0198 (3)	0.0839 (18)	
C3	0.3148 (4)	0.0426 (9)	0.7842 (4)	0.0532 (14)	
H3A	0.3390	−0.0242	0.7331	0.064*	
H3B	0.2700	−0.0453	0.8126	0.064*	
C4	0.2447 (4)	0.2185 (7)	0.7446 (4)	0.0459 (13)	
H4A	0.2342	0.2910	0.8003	0.055*	
O4	0.8467 (4)	0.5151 (11)	0.8999 (4)	0.110 (2)	
C5	0.1243 (5)	0.1684 (10)	0.6886 (5)	0.0681 (17)	
O5	0.8772 (5)	−0.0458 (10)	0.9037 (4)	0.105	
H5A	0.8826	−0.1584	0.8822	0.126*	
C6	0.0641 (5)	0.3588 (11)	0.6566 (6)	0.077 (2)	
H6A	−0.0087	0.3302	0.6176	0.093*	
H6B	0.0525	0.4278	0.7121	0.093*	
C7	0.1256 (6)	0.4859 (10)	0.6020 (5)	0.074 (2)	
H7A	0.0842	0.6043	0.5856	0.089*	
H7B	0.1322	0.4224	0.5437	0.089*	
C8	0.2398 (5)	0.5316 (9)	0.6592 (4)	0.0623 (17)	
H8A	0.2318	0.6051	0.7145	0.075*	
H8B	0.2780	0.6123	0.6215	0.075*	
C9	0.3132 (5)	0.3529 (8)	0.6926 (3)	0.0448 (13)	
C10	0.4166 (4)	0.4214 (7)	0.7671 (3)	0.0381 (11)	
H10A	0.3871	0.4927	0.8150	0.046*	
C11	0.4956 (5)	0.5621 (8)	0.7305 (4)	0.0577 (15)	
H11A	0.4749	0.5689	0.6620	0.069*	
H11B	0.4877	0.6897	0.7554	0.069*	
C12	0.6177 (5)	0.4975 (9)	0.7605 (4)	0.0587 (16)	
H12A	0.6669	0.5834	0.7344	0.070*	
C13	0.6456 (5)	0.5035 (10)	0.8703 (4)	0.0613 (16)	
H13A	0.6325	0.6331	0.8924	0.074*	
C14	0.5700 (4)	0.3595 (8)	0.9058 (3)	0.0413 (12)	
H14A	0.5249	0.4292	0.9432	0.050*	
C15	0.5630 (4)	0.1723 (7)	0.7609 (3)	0.0374 (11)	
H15A	0.5616	0.0424	0.7455	0.045*	
C16	0.6300 (5)	0.2951 (10)	0.7306 (4)	0.0539 (15)	
C17	0.7128 (7)	0.2472 (14)	0.6679 (5)	0.083 (2)	
H17A	0.7866	0.2892	0.7019	0.099*	
C18	0.6885 (7)	0.3491 (16)	0.5733 (6)	0.098	
H18A	0.6811	0.4845	0.5831	0.148*	
H18B	0.6205	0.3002	0.5352	0.148*	
H18C	0.7487	0.3271	0.5415	0.148*	
C19	0.7198 (7)	0.0228 (16)	0.6504 (6)	0.103 (3)	
H19A	0.7720	−0.0013	0.6107	0.155*	
H19B	0.6477	−0.0243	0.6198	0.155*	
H19C	0.7441	−0.0416	0.7099	0.155*	
C20	0.1193 (7)	0.0313 (11)	0.6079 (6)	0.096 (3)	
H20A	0.1590	−0.0841	0.6306	0.144*	
H20B	0.1527	0.0896	0.5605	0.144*	
H20C	0.0429	0.0005	0.5810	0.144*	
C21	0.0670 (7)	0.0619 (15)	0.7521 (6)	0.088 (2)	
C22	0.3508 (5)	0.2610 (10)	0.6074 (3)	0.0609 (16)	
H22A	0.3927	0.3530	0.5797	0.091*	
H22B	0.2865	0.2218	0.5611	0.091*	
H22C	0.3966	0.1509	0.6284	0.091*	
C23	0.6475 (4)	0.2278 (10)	0.9707 (3)	0.0520 (15)	
C24	0.7590 (5)	0.4422 (11)	0.9121 (4)	0.0631 (17)	
C25	0.8560 (5)	0.1800 (12)	1.0236 (5)	0.070 (2)	
H25A	0.8528	0.1827	1.0901	0.084*	
H25B	0.9222	0.2498	1.0166	0.084*	
C26	0.8656 (7)	−0.0272 (14)	0.9932 (5)	0.090 (3)	
H26A	0.9292	−0.0860	1.0349	0.109*	
H26B	0.7995	−0.0966	1.0007	0.109*	

### Atomic displacement parameters (Å^2^)

**Table:**

	*U*^11^	*U*^22^	*U*^33^	*U*^12^	*U*^13^	*U*^23^
N	0.037 (2)	0.070 (3)	0.049 (2)	−0.010 (2)	0.0038 (17)	0.004 (3)
C1	0.046 (3)	0.034 (3)	0.028 (2)	0.002 (2)	0.0057 (18)	0.004 (2)
O1	0.124 (5)	0.110 (6)	0.137 (6)	−0.016 (5)	0.033 (4)	−0.014 (5)
C2	0.041 (2)	0.044 (3)	0.039 (2)	0.002 (2)	0.002 (2)	0.011 (2)
O2	0.116	0.114	0.114	−0.034	0.071	−0.018
O3	0.046 (2)	0.133 (5)	0.067 (3)	−0.014 (3)	0.0006 (18)	0.062 (3)
C3	0.050 (3)	0.044 (3)	0.060 (3)	0.004 (3)	−0.002 (2)	0.006 (3)
C4	0.051 (3)	0.028 (3)	0.053 (3)	0.005 (2)	−0.003 (2)	−0.001 (2)
O4	0.063 (3)	0.124 (5)	0.133 (5)	−0.031 (3)	0.000 (3)	0.052 (4)
C5	0.060 (3)	0.058 (4)	0.080 (4)	0.005 (3)	0.000 (3)	−0.004 (3)
O5	0.105	0.105	0.105	0.000	0.022	0.000
C6	0.058 (4)	0.058 (4)	0.100 (5)	0.014 (3)	−0.023 (4)	0.006 (4)
C7	0.078 (4)	0.047 (4)	0.076 (4)	0.018 (4)	−0.031 (4)	0.007 (4)
C8	0.084 (4)	0.033 (3)	0.059 (3)	0.018 (3)	−0.009 (3)	0.001 (3)
C9	0.060 (3)	0.031 (3)	0.038 (3)	0.010 (3)	−0.002 (2)	−0.003 (2)
C10	0.056 (3)	0.029 (2)	0.029 (2)	0.007 (2)	0.007 (2)	−0.003 (2)
C11	0.072 (4)	0.035 (3)	0.060 (3)	−0.002 (3)	0.001 (3)	0.010 (3)
C12	0.066 (4)	0.047 (3)	0.061 (4)	−0.018 (3)	0.011 (3)	0.011 (3)
C13	0.067 (4)	0.042 (3)	0.070 (4)	−0.002 (3)	0.002 (3)	−0.010 (3)
C14	0.044 (3)	0.053 (3)	0.027 (2)	0.002 (2)	0.0063 (19)	−0.009 (2)
C15	0.053 (3)	0.029 (2)	0.033 (2)	0.001 (2)	0.015 (2)	−0.003 (2)
C16	0.058 (3)	0.067 (4)	0.040 (3)	0.007 (3)	0.017 (2)	0.007 (3)
C17	0.088 (5)	0.108 (7)	0.067 (4)	0.010 (5)	0.051 (4)	0.005 (5)
C18	0.098	0.098	0.098	0.000	0.021	0.000
C19	0.095 (6)	0.130 (8)	0.096 (6)	0.024 (6)	0.044 (5)	−0.034 (6)
C20	0.081 (5)	0.049 (4)	0.136 (7)	0.003 (4)	−0.029 (5)	−0.017 (5)
C21	0.071 (4)	0.090 (6)	0.098 (5)	0.009 (4)	0.011 (4)	0.005 (4)
C22	0.087 (4)	0.058 (4)	0.030 (2)	0.014 (4)	−0.006 (2)	−0.007 (3)
C23	0.038 (3)	0.079 (5)	0.038 (3)	0.001 (3)	0.005 (2)	0.000 (3)
C24	0.055 (3)	0.069 (4)	0.063 (4)	−0.018 (3)	0.007 (3)	0.003 (3)
C25	0.038 (3)	0.100 (6)	0.064 (4)	−0.005 (3)	−0.004 (3)	0.012 (4)
C26	0.089 (5)	0.107 (7)	0.078 (5)	0.039 (5)	0.022 (4)	0.030 (5)

### Geometric parameters (Å, °)

**Table:**

N—C23	1.395 (6)	C10—C11	1.548 (7)
N—C24	1.407 (8)	C10—H10A	0.9800
N—C25	1.472 (7)	C11—C12	1.536 (8)
C1—C15	1.506 (6)	C11—H11A	0.9700
C1—C2	1.535 (7)	C11—H11B	0.9700
C1—C10	1.563 (7)	C12—C16	1.489 (9)
C1—C14	1.571 (6)	C12—C13	1.551 (8)
O1—C21	1.251 (12)	C12—H12A	0.9800
C2—C3	1.535 (7)	C13—C24	1.459 (9)
C2—H2A	0.9700	C13—C14	1.526 (8)
C2—H2B	0.9700	C13—H13A	0.9800
O2—C21	1.222 (10)	C14—C23	1.498 (8)
O2—H2C	0.8500	C14—H14A	0.9800
O3—C23	1.192 (8)	C15—C16	1.322 (8)
C3—C4	1.535 (7)	C15—H15A	0.9300
C3—H3A	0.9700	C16—C17	1.534 (8)
C3—H3B	0.9700	C17—C18	1.512 (11)
C4—C9	1.554 (8)	C17—C19	1.586 (14)
C4—C5	1.567 (8)	C17—H17A	0.9800
C4—H4A	0.9800	C18—H18A	0.9600
O4—C24	1.236 (7)	C18—H18B	0.9600
C5—C21	1.470 (11)	C18—H18C	0.9600
C5—C20	1.497 (10)	C19—H19A	0.9600
C5—C6	1.539 (9)	C19—H19B	0.9600
O5—C26	1.337 (9)	C19—H19C	0.9600
O5—H5A	0.8500	C20—H20A	0.9600
C6—C7	1.491 (10)	C20—H20B	0.9600
C6—H6A	0.9700	C20—H20C	0.9600
C6—H6B	0.9700	C22—H22A	0.9600
C7—C8	1.503 (8)	C22—H22B	0.9600
C7—H7A	0.9700	C22—H22C	0.9600
C7—H7B	0.9700	C25—C26	1.518 (13)
C8—C9	1.550 (7)	C25—H25A	0.9700
C8—H8A	0.9700	C25—H25B	0.9700
C8—H8B	0.9700	C26—H26A	0.9700
C9—C22	1.541 (7)	C26—H26B	0.9700
C9—C10	1.556 (7)		
			
C23—N—C24	110.5 (5)	C11—C12—C13	105.8 (5)
C23—N—C25	124.2 (5)	C16—C12—H12A	110.9
C24—N—C25	125.1 (5)	C11—C12—H12A	110.9
C15—C1—C2	113.9 (4)	C13—C12—H12A	110.9
C15—C1—C10	109.8 (3)	C24—C13—C14	105.3 (5)
C2—C1—C10	111.4 (4)	C24—C13—C12	113.6 (5)
C15—C1—C14	105.6 (4)	C14—C13—C12	107.9 (5)
C2—C1—C14	111.0 (4)	C24—C13—H13A	110.0
C10—C1—C14	104.6 (4)	C14—C13—H13A	110.0
C1—C2—C3	113.4 (4)	C12—C13—H13A	110.0
C1—C2—H2A	108.9	C23—C14—C13	105.1 (4)
C3—C2—H2A	108.9	C23—C14—C1	114.6 (5)
C1—C2—H2B	108.9	C13—C14—C1	111.8 (4)
C3—C2—H2B	108.9	C23—C14—H14A	108.4
H2A—C2—H2B	107.7	C13—C14—H14A	108.4
C21—O2—H2C	107.4	C1—C14—H14A	108.4
C4—C3—C2	109.9 (5)	C16—C15—C1	115.9 (5)
C4—C3—H3A	109.7	C16—C15—H15A	122.1
C2—C3—H3A	109.7	C1—C15—H15A	122.1
C4—C3—H3B	109.7	C15—C16—C12	113.9 (5)
C2—C3—H3B	109.7	C15—C16—C17	126.2 (7)
H3A—C3—H3B	108.2	C12—C16—C17	119.8 (6)
C3—C4—C9	110.0 (4)	C18—C17—C16	113.8 (7)
C3—C4—C5	114.1 (5)	C18—C17—C19	108.9 (7)
C9—C4—C5	115.5 (4)	C16—C17—C19	112.0 (7)
C3—C4—H4A	105.4	C18—C17—H17A	107.3
C9—C4—H4A	105.4	C16—C17—H17A	107.3
C5—C4—H4A	105.4	C19—C17—H17A	107.3
C21—C5—C20	102.6 (7)	C17—C18—H18A	109.5
C21—C5—C6	111.0 (6)	C17—C18—H18B	109.5
C20—C5—C6	112.1 (6)	H18A—C18—H18B	109.5
C21—C5—C4	108.2 (5)	C17—C18—H18C	109.5
C20—C5—C4	115.0 (6)	H18A—C18—H18C	109.5
C6—C5—C4	107.7 (5)	H18B—C18—H18C	109.5
C26—O5—H5A	118.4	C17—C19—H19A	109.5
C7—C6—C5	113.8 (6)	C17—C19—H19B	109.5
C7—C6—H6A	108.8	H19A—C19—H19B	109.5
C5—C6—H6A	108.8	C17—C19—H19C	109.5
C7—C6—H6B	108.8	H19A—C19—H19C	109.5
C5—C6—H6B	108.8	H19B—C19—H19C	109.5
H6A—C6—H6B	107.7	C5—C20—H20A	109.5
C6—C7—C8	110.6 (5)	C5—C20—H20B	109.5
C6—C7—H7A	109.5	H20A—C20—H20B	109.5
C8—C7—H7A	109.5	C5—C20—H20C	109.5
C6—C7—H7B	109.5	H20A—C20—H20C	109.5
C8—C7—H7B	109.5	H20B—C20—H20C	109.5
H7A—C7—H7B	108.1	O2—C21—O1	121.1 (9)
C7—C8—C9	114.5 (5)	O2—C21—C5	115.3 (9)
C7—C8—H8A	108.6	O1—C21—C5	123.6 (9)
C9—C8—H8A	108.6	C9—C22—H22A	109.5
C7—C8—H8B	108.6	C9—C22—H22B	109.5
C9—C8—H8B	108.6	H22A—C22—H22B	109.5
H8A—C8—H8B	107.6	C9—C22—H22C	109.5
C22—C9—C8	109.7 (4)	H22A—C22—H22C	109.5
C22—C9—C4	114.9 (5)	H22B—C22—H22C	109.5
C8—C9—C4	107.1 (4)	O3—C23—N	120.6 (5)
C22—C9—C10	109.9 (4)	O3—C23—C14	130.7 (5)
C8—C9—C10	107.8 (4)	N—C23—C14	108.7 (5)
C4—C9—C10	107.1 (4)	O4—C24—N	122.6 (6)
C11—C10—C9	115.8 (4)	O4—C24—C13	127.2 (7)
C11—C10—C1	107.8 (4)	N—C24—C13	110.2 (5)
C9—C10—C1	114.8 (4)	N—C25—C26	112.7 (6)
C11—C10—H10A	105.9	N—C25—H25A	109.1
C9—C10—H10A	105.9	C26—C25—H25A	109.1
C1—C10—H10A	105.9	N—C25—H25B	109.1
C12—C11—C10	111.3 (4)	C26—C25—H25B	109.1
C12—C11—H11A	109.4	H25A—C25—H25B	107.8
C10—C11—H11A	109.4	O5—C26—C25	113.7 (7)
C12—C11—H11B	109.4	O5—C26—H26A	108.8
C10—C11—H11B	109.4	C25—C26—H26A	108.8
H11A—C11—H11B	108.0	O5—C26—H26B	108.8
C16—C12—C11	110.3 (5)	C25—C26—H26B	108.8
C16—C12—C13	108.0 (5)	H26A—C26—H26B	107.7
			
C15—C1—C2—C3	−77.6 (5)	C12—C13—C14—C23	124.2 (5)
C10—C1—C2—C3	47.3 (6)	C24—C13—C14—C1	−122.3 (5)
C14—C1—C2—C3	163.3 (4)	C12—C13—C14—C1	−0.7 (6)
C1—C2—C3—C4	−55.8 (6)	C15—C1—C14—C23	−65.5 (5)
C2—C3—C4—C9	63.4 (6)	C2—C1—C14—C23	58.4 (5)
C2—C3—C4—C5	−164.9 (5)	C10—C1—C14—C23	178.7 (4)
C3—C4—C5—C21	58.1 (7)	C15—C1—C14—C13	54.0 (5)
C9—C4—C5—C21	−172.9 (6)	C2—C1—C14—C13	177.9 (5)
C3—C4—C5—C20	−55.9 (8)	C10—C1—C14—C13	−61.9 (5)
C9—C4—C5—C20	73.1 (7)	C2—C1—C15—C16	−177.4 (4)
C3—C4—C5—C6	178.3 (6)	C10—C1—C15—C16	56.9 (6)
C9—C4—C5—C6	−52.8 (7)	C14—C1—C15—C16	−55.3 (5)
C21—C5—C6—C7	172.6 (6)	C1—C15—C16—C12	−1.6 (7)
C20—C5—C6—C7	−73.3 (8)	C1—C15—C16—C17	−179.9 (5)
C4—C5—C6—C7	54.2 (8)	C11—C12—C16—C15	−54.7 (6)
C5—C6—C7—C8	−57.6 (8)	C13—C12—C16—C15	60.5 (7)
C6—C7—C8—C9	57.4 (8)	C11—C12—C16—C17	123.8 (6)
C7—C8—C9—C22	72.0 (7)	C13—C12—C16—C17	−121.0 (6)
C7—C8—C9—C4	−53.3 (7)	C15—C16—C17—C18	118.0 (8)
C7—C8—C9—C10	−168.3 (5)	C12—C16—C17—C18	−60.3 (9)
C3—C4—C9—C22	60.7 (6)	C15—C16—C17—C19	−6.0 (10)
C5—C4—C9—C22	−70.2 (6)	C12—C16—C17—C19	175.7 (7)
C3—C4—C9—C8	−177.1 (4)	C20—C5—C21—O2	−176.4 (7)
C5—C4—C9—C8	51.9 (6)	C6—C5—C21—O2	−56.5 (9)
C3—C4—C9—C10	−61.7 (5)	C4—C5—C21—O2	61.5 (9)
C5—C4—C9—C10	167.3 (5)	C20—C5—C21—O1	3.9 (10)
C22—C9—C10—C11	55.9 (6)	C6—C5—C21—O1	123.8 (9)
C8—C9—C10—C11	−63.6 (6)	C4—C5—C21—O1	−118.1 (9)
C4—C9—C10—C11	−178.6 (4)	C24—N—C23—O3	−174.9 (6)
C22—C9—C10—C1	−70.8 (5)	C25—N—C23—O3	0.2 (9)
C8—C9—C10—C1	169.7 (4)	C24—N—C23—C14	4.6 (6)
C4—C9—C10—C1	54.7 (5)	C25—N—C23—C14	179.6 (5)
C15—C1—C10—C11	−51.5 (5)	C13—C14—C23—O3	175.0 (7)
C2—C1—C10—C11	−178.7 (4)	C1—C14—C23—O3	−61.9 (8)
C14—C1—C10—C11	61.4 (5)	C13—C14—C23—N	−4.4 (6)
C15—C1—C10—C9	79.2 (5)	C1—C14—C23—N	118.7 (5)
C2—C1—C10—C9	−48.0 (5)	C23—N—C24—O4	179.3 (7)
C14—C1—C10—C9	−167.9 (4)	C25—N—C24—O4	4.3 (10)
C9—C10—C11—C12	−131.2 (5)	C23—N—C24—C13	−2.9 (7)
C1—C10—C11—C12	−1.1 (6)	C25—N—C24—C13	−177.9 (6)
C10—C11—C12—C16	53.9 (6)	C14—C13—C24—O4	177.7 (7)
C10—C11—C12—C13	−62.6 (6)	C12—C13—C24—O4	59.9 (10)
C16—C12—C13—C24	60.7 (7)	C14—C13—C24—N	0.0 (7)
C11—C12—C13—C24	178.9 (5)	C12—C13—C24—N	−117.9 (6)
C16—C12—C13—C14	−55.6 (6)	C23—N—C25—C26	66.0 (8)
C11—C12—C13—C14	62.5 (6)	C24—N—C25—C26	−119.6 (7)
C24—C13—C14—C23	2.6 (6)	N—C25—C26—O5	62.4 (9)

### Hydrogen-bond geometry (Å, °)

**Table:**

*D*—H···*A*	*D*—H	H···*A*	*D*···*A*	*D*—H···*A*
O2—H2c···O5^i^	0.85	2.16	3.010 (9)	178
O5—H5a···O4^ii^	0.85	2.34	3.076 (10)	145
C10—H10A···O3^iii^	0.98	2.55	3.470 (6)	157
C14—H14A···O3^iii^	0.98	2.38	3.316 (7)	159

Symmetry codes: (i) *x*−1, *y*, *z*; (ii) *x*, *y*−1, *z*; (iii) −*x*+1, *y*+1/2, −*z*+2.
